# Free Volume Structure of Acrylic-Type Dental Nanocomposites Tested with Annihilating Positrons

**DOI:** 10.1186/s11671-016-1751-8

**Published:** 2016-11-28

**Authors:** Olha Shpotyuk, Adam Ingram, Oleh Shpotyuk

**Affiliations:** 1 Danylo Halytsky Lviv National Medical University, 69, Pekarska str., Lviv, 79010 Ukraine; 2 Opole University of Technology, 75, Ozimska str., 45370 Opole, Poland; 3 Jan Dlugosz University in Czestochowa, 13/15, Armii Krajowej str., 42200 Czestochowa, Poland; 4 Vlokh Institute of Physical Optics, 23, Dragomanov str., Lviv, 79005 Ukraine

**Keywords:** Dental resin composites, Light curing, Positron annihilation, Positronium, Trapping

## Abstract

Positron annihilation spectroscopy in lifetime measuring mode exploring conventional fast-fast coincidence ORTEC system is employed to characterize free volume structure of commercially available acrylic-type dental restorative composite Charisma® (Heraeus Kulzer GmbH, Germany). The measured lifetime spectra for uncured and light-cured composites are reconstructed from unconstrained x3-term fitting and semi-empirical model exploring x3-x2-coupling decomposition algorithm. The governing channel of positron annihilation in the composites studied is ascribed to mixed positron-Ps trapping, where Ps decaying in the third component is caused entirely by input from free-volume holes in polymer matrix, while the second component is defined by free positron trapping in interfacial free-volume holes between filler nanoparticles and surrounded polymer matrix. Microstructure scenario of the photopolymerization shrinkage includes cross-linking of structural chains in polymer matrix followed by conversion of bound positron-electron (positronium) traps in positron-trapping interfacial free-volume voids in a vicinity of agglomerated filler nanoparticles.

## Background

Progress in the modern biomedicine is governed by successful development of novel materials effectively exploring nanostructural atomistic and sub-atomistic length scales. That is why high-informative characterization probes are needed to engineer such biomaterials suitable for advanced medical practice. One of the most promising probes is grounded on analytical space-time continuum determination for electron interaction with its antiparticle (positron), this instrumentation tool being termed as positron annihilation lifetime (PAL) spectroscopy [1–3].

In this work, the methodological possibilities of PAL technique will be analyzed and examined in application to light-cured acrylic-type dental resin composite (DRC) Charisma®.

## Methods

The Charisma® is commercially available micro-hybrid DRC produced by Heraeus Kulzer GmbH (Germany), possessing monomer matrix consisted of bisphenol A-diglycidyl dimethacrylate (BisGMA) and triethyleneglycol dimethacrylate (TEGDMA) modified with multi-sized filler fraction (near 78% by weight) composed of compact barium aluminum fluoride glass (radioopaque Ba-Al-B-F-Si Microglass®, d_50_ = 0.7 μm) and highly dispersive silicium dioxide SiO_2_ glass with finest ~10–20 nm nanoparticles [4, 5]. Because of this inhomogeneous nanometric constitution of filling system, such DRC reveal intrinsic free-volume void arrangement stretched down to sub-nanometer scale, i.e., character level of high sensitivity for PAL spectroscopy [1–3].

For our PAL measurements, the DRC Charisma® samples (in A3 shadow) were prepared by filling an inner volume of disk-shaped plastic molds of uniform size having 6 mm in diameter and 2 mm in thickness. Bottom-end surface of plastic disk was covered by polyethylene slice film, separated from DRC sample along with around-disk outer ring just before PAL experiments. This batch of non-polymerized (uncured) DRC samples of character paste-like consistency was marked as Charisma-0. Part of these DRC samples were then polymerized by illuminating their upper surfaces with standard curing dental wireless LED source (LED.T4, SEASKY, China) emitted light irradiation in 420–480 nm range with ~900 mW/cm^2^ output power density. To normalize the light-curing protocol, the end of the guide tip from light source was maintained just above the sample surface at a distance of 7 mm, so sample’s surface was fully covered by light beam. The light illumination durations were 5, 20, and 60 s, the latter exposure ensuring deeply polymerized DRC Charisma® state (in respect to the manufacturers’ manual [4, 5]). The light-cured DRC samples marked as Charisma-1,2,3 for different curing durations (5, 20, and 60 s, respectively) were then stored dry at room temperature for 3 days before PAL measurements.

The PAL spectra were registered with a fast-fast coincidence system of 230 ps resolution based on two Photonis XP2020/Q photomultiplier tubes coupled to BaF_2_ scintillator 25.4A10/2M-Q-BaF-X-N detectors (Scionix, Bunnik, Holland) and ORTEC® electronics (ORTEC, Oak Ridge, TN, USA) [3]. To ensure reliable measurements, each PAL spectrum was recorded at high stabilized temperature of *T* = 22 °C and relative humidity of 35% in a normal-measurement statistics reaching nearly 1 million of coincidences. The channel width of 6.15 ps allows a total number of channels to be 8000. The radioactive ^22^Na isotope of low ~50 kBq activity was used as source of positrons sandwiched between two tested DRC samples.

The measured PAL spectra were processed with LT 9.0 program [6], stabilizing an average positron lifetime as a center of mass of full PAL spectrum:

1 $$ {\tau}_{\mathrm{av}}^{\varSigma }={\displaystyle \sum_i{I}_i{\tau}_i} $$

where *τ*_*i*_ and *I*_*i*_ denote lifetime and intensity of the corresponding fitting components.

The resulting accuracy in positron lifetimes *τ*_*i*_ and intensities *I*_*i*_ under above spectrometer resolution was not worse ±0.005 ns and 0.5%, respectively.

The best fitting of the PAL spectra was achieved via mixed channels of trapping, which occurs through defect-related positron trapping sites and bound positron-electron (positronium Ps) states. This task can be solved due to multi-component fitting of PAL spectra with three or four negative exponentials. Because of repulsive interaction between positron and atomic nuclei of environment, positron samples intrinsic regions of minimal positive charge density, preferentially negative-charged or neutral free-volume voids. Describing positron trapping in terms of two-state model with only one kind of defects, the parameters of defect-free bulk lifetime *τ*_*b*_, trapping rate in defects *κ*_*d*_, and fraction of trapped positrons *η* can be simply calculated in respect to known formalism [1, 7, 8]. In addition, the difference between defect-related and defect-free lifetimes (*τ*_*2*_–*τ*_*b*_) can be accepted as a signature of size of positron traps in terms of equivalent number of vacancies [1].

Other channel is caused by positron annihilation from Ps state as free particles or interacting with an electron from environment [1, 2, 9]. In the ground state, Ps exists as para-Ps (p-Ps, antiparallel positron-electron spins) decaying intrinsically with two γ-quanta and character 0.125 ns lifetime in a vacuum and ortho-Ps (o-Ps, parallel spins) decaying with three γ-quanta and 142 ns lifetime, these states being occupied with 1:3 ratio. In a matter, since positron wave function is overlapping with electron outside, the annihilation with such electron having an antiparallel spin decreases lifetime to 0.5–10 ns resulting in two γ-rays (“pick-off” annihilation). The Ps localized in free-volume spaces gives indication on their mean radii *R* in terms of long-lived *τ*_3_ lifetime (relative intensity of this component *I*_3_ correlates with density of Ps sites) in respect to Tao-Eldrup equation:

2 $$ {\tau}_{\mathbf{3}}=0.5\cdot {\left[1-\frac{R}{R+\varDelta R}+\frac{1}{2\pi}\cdot \sin \left(\frac{2\pi R}{R+\varDelta R}\right)\right]}^{-1} $$

where Δ*R* = 0.166 nm is fitted empirical layer thickness [1, 2].

By fitting Eq. (2) with measured *τ*_3_ value, the radius *R*_3_ and spherical free volumes *V*_*f*_ can be determined, giving a possibility to calculate fractional free volume using empirical constant *C* = 0.0018 Å^−3^ [9].

## Results and Discussion

**Fig. 1:**
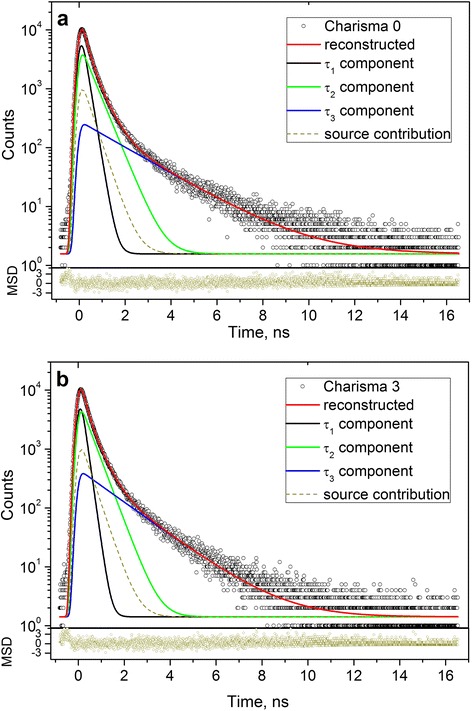
PAL spectra of non-polymerized Charisma-0 (**a**) and polymerized Charisma-3 (**b**) composites reconstructed from unconstrained x3-fitting procedure at the general background of source contribution. The *bottom insets* show statistical scatter of variance

**Fig. 2:**
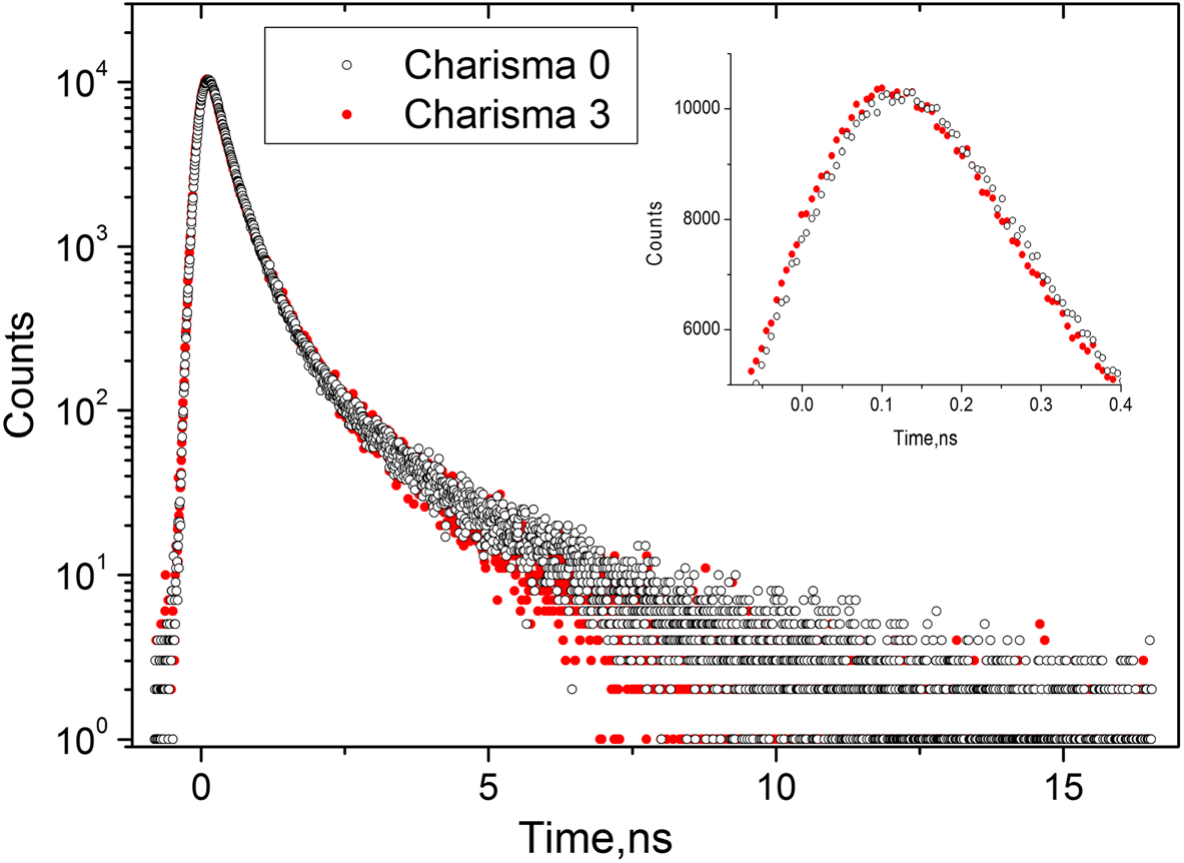
PAL spectra of non-polymerized Charisma-0 (*black open circles*) and polymerized Charisma-3 (*red full circles*) composites in *semi-log* presentation. The *inset* shows linearized comparison of annihilation events accumulated in the peak

The measured raw PAL spectra of DRC Charisma® in Fig. 1a, b depicted for initial and fully polymerized (cured during 60 s) states at the general background of source contribution, respectively, were reconstructed from unconstrained x3-fitting route. The narrow-restricted statistical scatter of variance tightly grouped around 0-axis testifies that PAL measurements was adequately described within this decomposition, best-fit parameters, positron, and Ps-trapping modes being gathered in Table 1. Attempt to decompose these spectra on four components exploring partially constrained x4-fitting route (under shortest lifetime fixed as intrinsic p-Ps self-annihilation lifetime τ_1_ = 0.125 ns) is not followed by essential improvement in a goodness. Comparison of PAL spectra for non-polymerized Charisma-0 and fully polymerized Charisma-3 DRC is given in Fig. 2. It is clearly seen that more sharp slope in the edge of PAL spectra for light-cured Charisma-3 is well attributed to reduced *τ*_3_ lifetimes (Table 1), while left shift in the top of annihilating events (insert to Fig. 2) testifies in a favor of smaller *τ*_1_ lifetimes in these DRC.

**Table 1:** The best-fit PAL spectra parameters and trapping modes for DRC Charisma® determined within unconstrained x3-decomposion procedure

Sample	PAL spectra fitting parameters						Positron trapping modes				Ps-trapping modes	
	τ_1_, ns	τ_2_, ns	τ_3_, ns	I_2_, a.u.	I_3_, a.u.	τ_av._, ns	τ_b_, ns	κ_d_, ns^−1^	τ_2_-τ_b_, ns	η, a.u.	R_3_, nm	f_v_^3^, %
0	0.179	0.447	1.946	0.540	0.092	0.486	0.277	1.99	0.170	0.36	0.283	1.58
1	0.168	0.435	1.747	0.550	0.105	0.479	0.269	2.22	0.166	0.37	0.263	1.44
2	0.162	0.421	1.625	0.550	0.106	0.461	0.262	2.33	0.159	0.38	0.250	1.25
3	0.158	0.415	1.560	0.560	0.110	0.458	0.260	2.50	0.155	0.40	0.243	1.19

Numerous experiments prove that x3-term analysis is most commonly applied to reconstruct the PAL spectra for structurally inhomogeneous polymer/filler nanocomposites like DRC [10–17]. Under such analysis (performed as unconstrained decomposition [10–17] or partially constrained decomposition fixing shortest *τ*_1_ lifetime [10, 11, 17]), the long-lived component is attributed to o-Ps annihilation in free-volume holes, the second component with intermediate lifetime *τ*_2_ is due to free positron annihilation in interfacial free volumes or other defect states in solid phase, and the shortest-lived component is ascribed to p-Ps self-annihilation overlapped with reduced positron annihilation from defect-free bulk states [1, 2]. The lifetime fixing is preferred to overcome inadequacy in the resolving of first shortest component due to mixing different annihilation events (especially, when *I*_1_ occurs to be substantially greater than *I*_3_) [10, 11]. In such a case, fixing *τ*_1_ to the value of p-Ps lifetime improves the reliability of finite-term analysis, not affecting the value of o-Ps lifetime [10]. Nevertheless, unconstrained x3-decomposition has some, albeit limited, physical relevance allowing most stable fitting procedure [10]. Our previous results with some acrylic-type DRC [16] also show that even under incomplete decomposition of PAL spectra because of some measuring instabilities, in part, in a vicinity of second component originated from free positron annihilation, the best goodness of PAL spectra reconstruction is achieved under constraint-free x3-fitting.

In respect to the PAL data parameterized within free x3-term analysis (Table 1), the annihilation process in all DRC Charisma® samples can be identified as mixed positron-Ps trapping, where “pure” o-Ps decaying is caused entirely by input from free-volume holes in polymer matrix (third component), while the second component is defined mainly by cumulative input of free positron-trapping sites in a filler (including interfacial free-volume holes between filler nanoparticles). Typical radii of o-Ps-traps in the DRC estimated from Eq. (2) slightly scatter approaching 3 Å (0.283 nm in Charisma-0 and 0.243 nm in Charisma-3). The same concerns positron-trapping channel defined by defect lifetimes *τ*_2_ ≅ 0.42–0.45 ns (strict parameterization of this channel in term of two-state trapping model [1–3, 7, 8] is rather meaningless under essential input of o-Ps decay).

It is well known that light curing causes volumetric shrinkage in polymer/filler composites, this macroscopic-sensitive parameter approaching 2.9% for DRC Charisma® [4, 5]. Due to trapping parameters defined from x3-component best-fitted PAL spectra (Table 1), photopolymerization shrinkage in the studied Charisma® DRC is well revealed through decrease in average lifetime of annihilating positrons *τ*_av*.*_ (from 0.486 ns in Charisma-0 to 0.458 ns in Charisma-3, i.e., on 5.8%), this effect being caused by mutually opposite changes in o-Ps and positron trapping channels. The photopolymerized DRC possess reduced long-lived *τ*_3_ lifetimes, but increased *I*_3_ intensities, thus resulting in smaller fractional free volumes *f*_v_ (on ~25%). At the same time, the fraction of trapped positrons *η* shows over-balanced tendency with light curing due to increase on ~28% (from 0.36 in Charisma-0 to 0.40 in Charisma-3), resulting from higher *I*_2_ intensities but suppressed *τ*_2_ lifetimes for light-cured DRC Charisma® (Table 1). Noteworthy, these parameters change monotonically with photoexposure tending to obvious saturation after 60 s of light curing.

Changes in Ps-trapping modes can be ascribed preferentially to cross-linking of structural chains dominated in pure polymer matrix, like it occurs in other polymers under UV light exposure [18], vulcanization (thermal curing) [19], or γ-irradiation [20]. In fact, this photoinduced process causes smaller voids in DRC owing to overall *free-volume fragmentation* in both o-Ps and positron trapping sites. In a vicinity of agglomerated filler particles, the fragmented o-Ps traps convert in *interfacial voids* (triple junctions or pseudogap holes at the interface between outer surface layer of filler particles and innermost layer of surrounding polymer matrix), which are also efficient trapping sites for positrons (corresponding to increased fraction of trapped positrons *η* in light-cured DRC).

By assuming full conversion of o-Ps trapping sites in interfacial positron traps (ignoring void fragmentation without changing in trapping type), we can treat these data within x3-x2-coupling decomposition algorithm [21, 22]. Within this approach, we deal with x3-term PAL spectrum transformed to generalized x2-term form for host (non-polymerized or uncured) and modified (light-cured) DRC matrix (second component involves contributions from all trapping channels, including positron traps, input from o-Ps decaying, and p-Ps self-annihilation). This allows resolving additional input with lifetime *τ*_int_ and intensity *I*_int_ in second component of generalized x2-term PAL spectrum for modified matrix, compensating (*τ*_n_,*I*_n_) input in first channel being found assuming full inter-channel equilibrium. Thereby, parameterization of transformed Ps-positron traps in modified DRC matrix can be performed by accepting (*τ*_n_,*I*_n_) and (*τ*_int_,*I*_int_) as first and second components of x2-term PAL spectrum for hypothetical media obeying parameterization in respect to formalism of conventional two-state trapping model [1–3, 7, 8]. Since preferential disappearing of free-volume voids under light-curing polymerization in polymer/filler composite systems, this mathematical treatment of experimental x3-term decomposed PAL spectra is conveniently to perform for uncured sample in respect to light-cured ones (in this case, both *I*_n_ and *I*_int_ intensities are positive).

The PAL trapping parameters for light-cured DRC Charisma-1,2,3 determined in respect to non-polymerized Charisma-0 using x3-x2-coupling decomposition algorithm are presented in Table 2. Since higher *τ*_int_ values exceed a characteristic level of o-p-Ps self-annihilation in a vacuum (0.5 ns) [1, 2], the hypothetical medium formed in the DRC under photopolymerization can be imagined as rather loose substance possessing a wide range of o-Ps trapping sites. Just these free-volume holes with radii of 0.130 nm estimated in respect to Eq. (2) disappear under light curing from DRC matrix on a cost of interfacial positron traps near agglomerated filler nanoparticles.

**Table 2:** PAL trapping modes of uncured DRC Charisma-0 in respect to light-cured DRC Charisma-1,2,3 samples calculated within x3-x2-coupling decomposition algorithm [21, 22]

DRC	First component		Second component		Trapping modes		
	τ_n_, ns	I_n_, a.u.	τ_int_, ns	I_int_, a.u.	τ_av_, ns	τ_b_, ns	κ_d_, ns^−1^
Charisma-1	0.319	0.026	0.547	0.058	0.477	0.449	0.90
Charisma-2	0.388	0.027	0.645	0.063	0.568	0.538	0.72
Charisma-3	0.409	0.030	0.656	0.072	0.584	0.558	0.65

## Conclusions

Characterization possibilities of positron annihilation lifetime (PAL) spectroscopy is analyzed in application to commercially available acrylic-type dental restorative composite Charisma® (Heraeus Kulzer GmbH, Germany) subjected to light curing during 5, 20, and 60 s from LED source emitted in 420–480 nm range with ~900 mW/cm^2^ power density.

In respect to the PAL data parameterized within unconstrained x3-term analysis, the governing annihilation process in the Charisma® composite is identified as mixed positron-Ps trapping, where Ps decaying in the third component is caused entirely by input from free-volume holes in polymer matrix, while the second component is defined by free positron trapping in interfacial free-volume holes between filler nanoparticles and surrounded polymer matrix.

The photopolymerization shrinkage in the Charisma® composites is revealed through decrease in average lifetime of annihilating positrons due to mutually opposite changes in Ps and positron annihilation channels. The growing light-activated polymerization kinetics is detected for both intensities related to second and third components in the x3-term decomposed lifetime spectra accompanied by decrease in the corresponding positron lifetimes, this process resulting in essentially enhanced trapping rate in defects and depressed fractional free volume saturated with light curing. Light exposure causes smaller voids in composites owing to free-volume fragmentation in Ps- and positron trapping sites. Plausible microstructure scenario for these transformations includes photo-induced cross-linking of structural chains in polymer matrix followed by conversion of o-Ps traps in interfacial free-volume voids in a vicinity of agglomerated filler nanoparticles. Meaningful phenomenological description of this process can be developed at the basis of semi-empirical model exploring x3-x2-coupling decomposition algorithm.

## Abbreviations

BisGMA: Bisphenol A-diglycidyl dimethacrylate
DRC: Dental resin composite
PAL: Positron annihilation lifetime
TEGDMA: Triethyleneglycol dimethacrylate.
